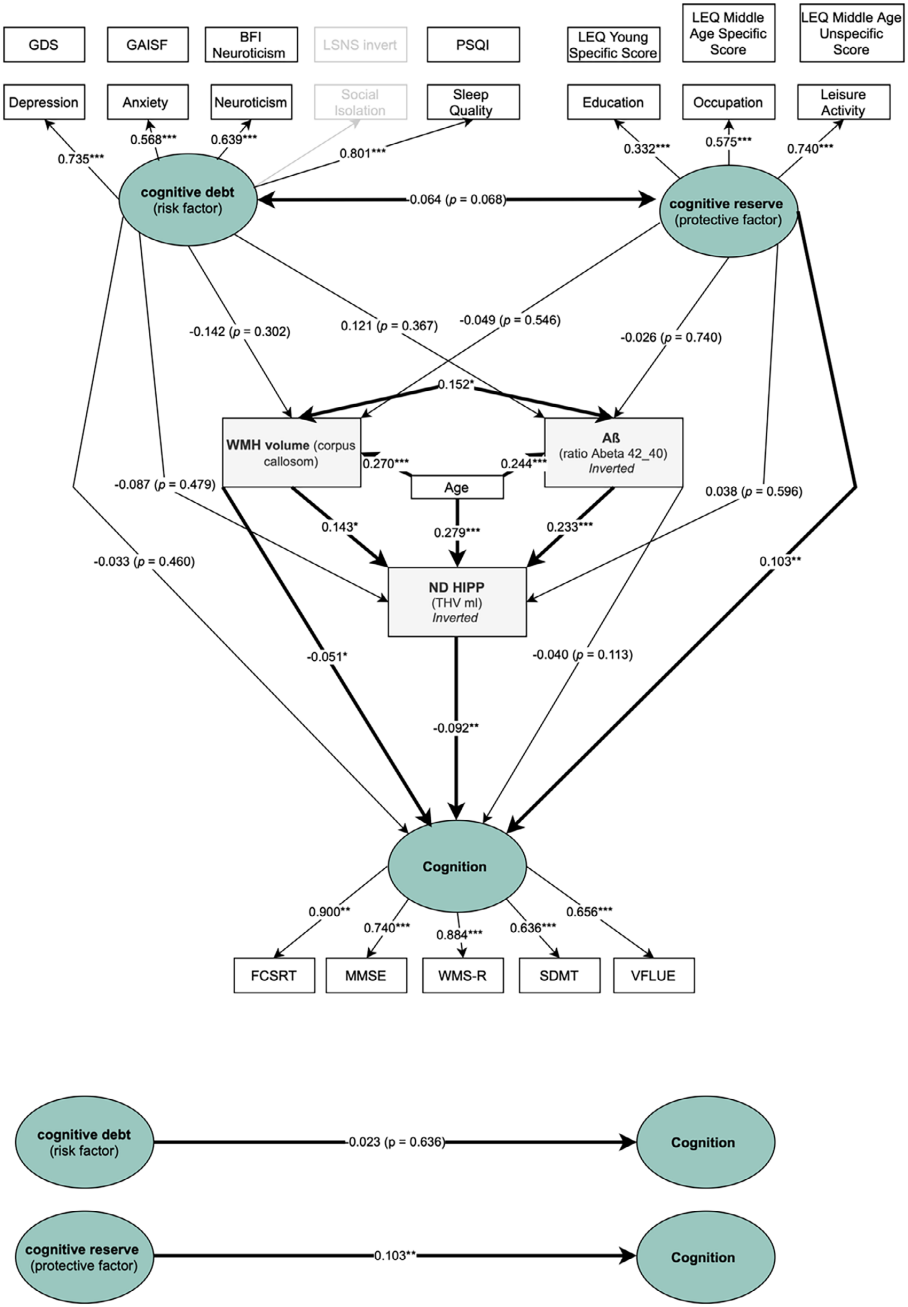# Associations of cognitive debt and cognitive reserve with mixed brain pathology and cognition

**DOI:** 10.1002/alz70856_102595

**Published:** 2025-12-25

**Authors:** Hanna Boscheck, Maxie Liebscher, Ylenia D'Elia, René Mauer, Oliver Peters, Josef Priller, Anja Schneider, Jens Wiltfang, Katharina Buerger, Robert Perneczky, Stefan Teipel, Christoph Laske, Annika Spottke, Frederic Brosseron, Renat Yakupov, Gabriel Ziegler, Luca Kleineidam, Frank Jessen, Emrah Düzel, Michael Wagner, Sandra Roeske, Natalie L Marchant, Franka Gloeckner, Olga M. Klimecki, Miranka Wirth, DELCODE study group

**Affiliations:** ^1^ German Center for Neurodegenerative Diseases (DZNE), Dresden, Germany; ^2^ Center for the Neuroscience of Embodied Cognition (CeNEC), California, USA; ^3^ Medical Faculty Carl Gustav Carus, Dresden, Germany; ^4^ German Center for Neurodegenerative Diseases (DZNE), Berlin, Germany; ^5^ Charité – Universitätsmedizin Berlin, corporate member of Freie Universität Berlin and Humboldt‐Universität zu Berlin, Berlin, Germany; ^6^ ECRC Experimental and Clinical Research Center, Charité Universitätsmedizin Berlin, Berlin, Germany; ^7^ University of Edinburgh and UK DRI, Edinburgh, United Kingdom; ^8^ Department of Psychiatry and Psychotherapy, School of Medicine and Health, Technical University of Munich, and German Center for Mental Health (DZPG), Munich, Germany; ^9^ Department of Psychiatry and Psychotherapy, Charité, Charitéplatz 1, Berlin, Germany; ^10^ Department of Old Age Psychiatry and Cognitive Disorders, University Hospital Bonn and University of Bonn, Bonn, Germany; ^11^ University Hospital Bonn and University of Bonn, Bonn, Germany; ^12^ German Center for Neurodegenerative Diseases (DZNE), Goettingen, Germany; ^13^ Department of Psychiatry and Psychotherapy, University Medical Center Goettingen, University of Goettingen, Goettingen, Germany; ^14^ Neurosciences and Signaling Group, Institute of Biomedicine (iBiMED), Department of Medical Sciences, University of Aveiro, Aveiro, Portugal; ^15^ Institute for Stroke and Dementia Research (ISD), University Hospital, LMU Munich, Munich, Germany; ^16^ German Center for Neurodegenerative Diseases (DZNE), Munich, Germany; ^17^ Ageing Epidemiology (AGE) Research Unit, School of Public Health, Imperial College London, London, United Kingdom; ^18^ Department of Neuroradiology, University Hospital LMU, Munich, Germany; ^19^ Munich Cluster for Systems Neurology (SyNergy), Munich, Germany; ^20^ German Center for Neurodegenerative Diseases (DZNE), Rostock, MV, Germany; ^21^ Department of Psychosomatic Medicine, University of Rostock, Rostock, Germany; ^22^ Section for Dementia Research, Hertie Institute for Clinical Brain Research and Department of Psychiatry and Psychotherapy, University of Tübingen, Tübingen, Germany; ^23^ German Center for Neurodegenerative Diseases (DZNE), Tübingen, Germany; ^24^ Department of Neurology, University of Bonn, Bonn, Germany; ^25^ German Center for Neurodegenerative Diseases (DZNE), Venusberg‐Campus 1, 53127, Bonn, Germany; ^26^ German Center for Neurodegenerative Diseases (DZNE), Bonn, Germany; ^27^ German Center for Neurodegenerative Diseases (DZNE), Magdeburg, Germany; ^28^ Institute of Cognitive Neurology and Dementia Research (IKND), Otto‐von‐Guericke University, Magdeburg, Germany; ^29^ Deutsches Zentrum für Neurodegenerative Erkrankungen e. V. (DZNE) Bonn, Bonn, Germany; ^30^ Department of Psychiatry, University of Cologne, Medical Faculty, Kerpener Strasse 62, Cologne, Germany; ^31^ Excellence Cluster on Cellular Stress Responses in Aging‐Associated Diseases (CECAD), Faculty of Medicine and University Hospital Cologne, Cologne, Germany; ^32^ Institute of Cognitive Neurology and Dementia Research (IKND), Otto‐von‐Guericke University, Magdeburg, Sachsen Anhalt, Germany; ^33^ University of Bonn Medical Center, Bonn, Germany; ^34^ University College London, London, United Kingdom; ^35^ Technische Universität Dresden, Dresden, Germany

## Abstract

**Background:**

Behavioral risk or protective factors related to “cognitive debt” and “cognitive reserve” may influence brain pathology and cognition and thereby contribute to resilience in aging. This cross‐sectional study examined direct and indirect associations of cognitive debt (risk factor) and cognitive reserve (protective factor) with mixed brain pathologies and cognition in older adults.

**Methods:**

A sample of *N* = 298 non‐demented older adults (mean age=70 years, 56% male) from the DELCODE study (DRKS00007966) were analyzed using structural equation modeling (SEM) and an a‐priori path model. We assessed the association between cognitive debt and cognitive reserve (modelled as latent constructs) and global cognition (Preclinical Alzheimer Cognitive Composite 5 [PACC5] modelled as latent construct) through pathological pathways involving beta‐amyloid (Aß) burden, hippocampal neurodegeneration, and white matter hyperintensities (WMH) in the corpus callosum splenium (CCs), while adjusting for age. A goodness‐of‐fit analysis ensured adequate model fit.

**Results:**

Brain pathology was associated with lower PACC5 performance via direct pathways (for WMH in the CCs and hippocampal neurodegeneration) and indirect pathways (for Aß deposition via hippocampal neurodegeneration) (all *p* < .05). Cognitive debt and cognitive reserve were not significantly associated with brain pathology (all *p* > .05). Cognitive reserve, but not cognitive debt, was independently associated with better PACC5 performance (*p* = .005). Cognitive debt and cognitive reserve were associated at trend level (*p* = .068). Results are displayed in Figure 1.

**Conclusion:**

Brain pathologies were linked to lower cognitive performance. Cognitive reserve, but not cognitive debt, was independently associated with better cognitive performance (1). There were no significant associations of cognitive debt and cognitive reserve with brain pathologies. The findings suggest that cognitive reserve may influence resilience through mechanisms independent of brain pathology (2). Future longitudinal studies are needed to investigate these pathways and clarify causal relationships.

**References**

1. Vemuri P, Weigand SD, Przybelski SA, Knopman DS, Smith GE, Trojanowski JQ, et al. Cognitive reserve and Alzheimer's disease biomarkers are independent determinants of cognition. Brain. 2011;134(Pt 5):1479‐92.

2. Vemuri P, Lesnick TG, Przybelski SA, Knopman DS, Roberts RO, Lowe VJ, et al. Effect of lifestyle activities on Alzheimer disease biomarkers and cognition. Ann Neurol. 2012;72(5):730‐8.